# MicroRNA-423-5p facilitates hypoxia/reoxygenation-induced apoptosis in renal proximal tubular epithelial cells by targeting GSTM1 via endoplasmic reticulum stress

**DOI:** 10.18632/oncotarget.18289

**Published:** 2017-05-30

**Authors:** Xiao-Peng Yuan, Long-Shan Liu, Chuan-Bao Chen, Jian Zhou, Yi-Tao Zheng, Xiao-Ping Wang, Ming Han, Chang-Xi Wang

**Affiliations:** ^1^ 3rd Division of Organ Transplant Center, Eastern Campus of The First Affiliated Hospital, Sun Yat-sen University, Guangzhou 510700, P.R. China; ^2^ 2nd Division of Organ Transplant Center, The First Affiliated Hospital, Sun Yat-sen University, Guangzhou 510080, P.R. China

**Keywords:** MicroRNA-423-5p, GSTM1, renal proximal tubule epithelial cells, epithelial cell repair, endoplasmic reticulum stress

## Abstract

It has been reported that microRNAs (miRs) can regulate renal response to acute injury and members of them are believed to be important in maintenance of renal function and development of renal injury. We investigated the actions of microRNA-423-5p (miR-423-5p) and glutathione-S-transferase (GST) M1 after acute kidney injury. MiR-423-5p was up-regulated and GSTM1 was down-regulated in human kidney (HK-2) cells subjected to hypoxia/reoxygenation (H/R) and in rat kidneys subjected to ischemia/reperfusion (I/R) injury. Dual luciferase assays revealed miR-423-5p binding to the 3′ untranslated region of GSTM1. Proliferation was lower and apoptosis, ER stress and oxidative stress were all higher in H/R-treated HK-2 cells transfected with or without miR-423-5p mimics and GSTM1 siRNA than in the same cells transfected with miR-423-5p inhibitors and a GSTM1 expression vector. Increased miR-423-5p and decreased GSTM1 mRNA and protein levels were observed in rat kidneys on days 1, 2 and 7 after I/R. Levels had normalized by days 14 and 21. On day 3 after treatment, rats receiving I/R or I/R plus miR-423-5p mimics exhibited higher serum creatinine and urea nitrogen levels than rats receiving I/R plus a miR-423-5p inhibitor. MiR-423-5p and lower GSTM1 mRNA and protein levels were higher in the I/R and I/R plus miR-423-5p mimic groups than in the I/R plus miR-423-5p inhibitors group. These findings demonstrate that after acute kidney injury, miR-423-5p induces ER stress and oxidative stress by inhibiting GSTM1and suppresses repair.

## INTRODUCTION

Renal proximal tubular epithelial cells (RPTECs) account for 90% of kidney volume and represent the major cell type constituting the entire tubulointerstitium [1]. They are vital for kidney function and any injury to RPTECs results in accumulation of myofibroblasts leading to renal tubulointerstitial fibrosis [2]. RPTEC injury is also a major risk factor for crystal formation in the kidneys [3]. RPTEC injury results in endoplasmic reticulum (ER) and oxidative stress, and ER is a site for membrane and protein biogenesis and folding [4]. Perturbations in ER results in accumulation of mis-folded proteins in the ER leading to ER stress [5]. The unfolded protein response (UPR) is induced in response to ER stress. Inefficient response to ER stress can result in cell death [6]. Renal damage can also occur due to oxidative damage that is a result in increased cellular oxidants due to formation of toxic reactive intermediates that damage cellular components like proteins, genome and the membranes [7]. Thus, identification of factors regulating ER and oxidative stresses are potentially promising for repairing RPTEC injury.

MicroRNAs (miRs) are a large family of non-coding RNAs, 19 to 25 nucleotides in length, which bind to the 3′-UTR of their target mRNAs, thereby regulating gene expression [8]. MiRs are involved in regulating a number of cellular processes. For example, a study on obesity revealed that miRs regulated oxidative stress, apoptosis and angiogenesis [9]. The miR-423-5p levels determined tumor cell growth in gastric cancer and predicted heart failure [10, 11]. Also, miR-199a-3p, miR-let-7i-5p and miR-423-3p are involved in maintaining renal function and response to renal injury [12].

GSTM1 encodes the glutathione-S-transferase (GST) M1 enzyme that is involved in detoxification of various carcinogens in lung cancer [13]. During renal tubular injury, various GST isoforms are released into the urine and can be detected earlier than changes in serum creatinine levels. Moreover, GSTs including GSTM1, GSTM3 and GSTT1 play a critical role in protecting cells from oxidative stress [14]. Loss of *GSTM1* gene could inhibit kidney disease progression; and GSTM1 and GSTT1 prevent renal cell injury due to carcinogens [15, 16]. GSTM1 is the most abundant GST enzyme in the mouse kidneys [17]. But, if GSTM1 is regulated by miR-423-5p is not known. Therefore, we investigated the relationship between the miR-423-5p and GSTM1 and their role in RPTEC repair after acute kidney injury.

## RESULTS

### Increased miRNA-423-5p expression in H/R induced HK-2 cells and I/R treated rats

**Figure 1:**
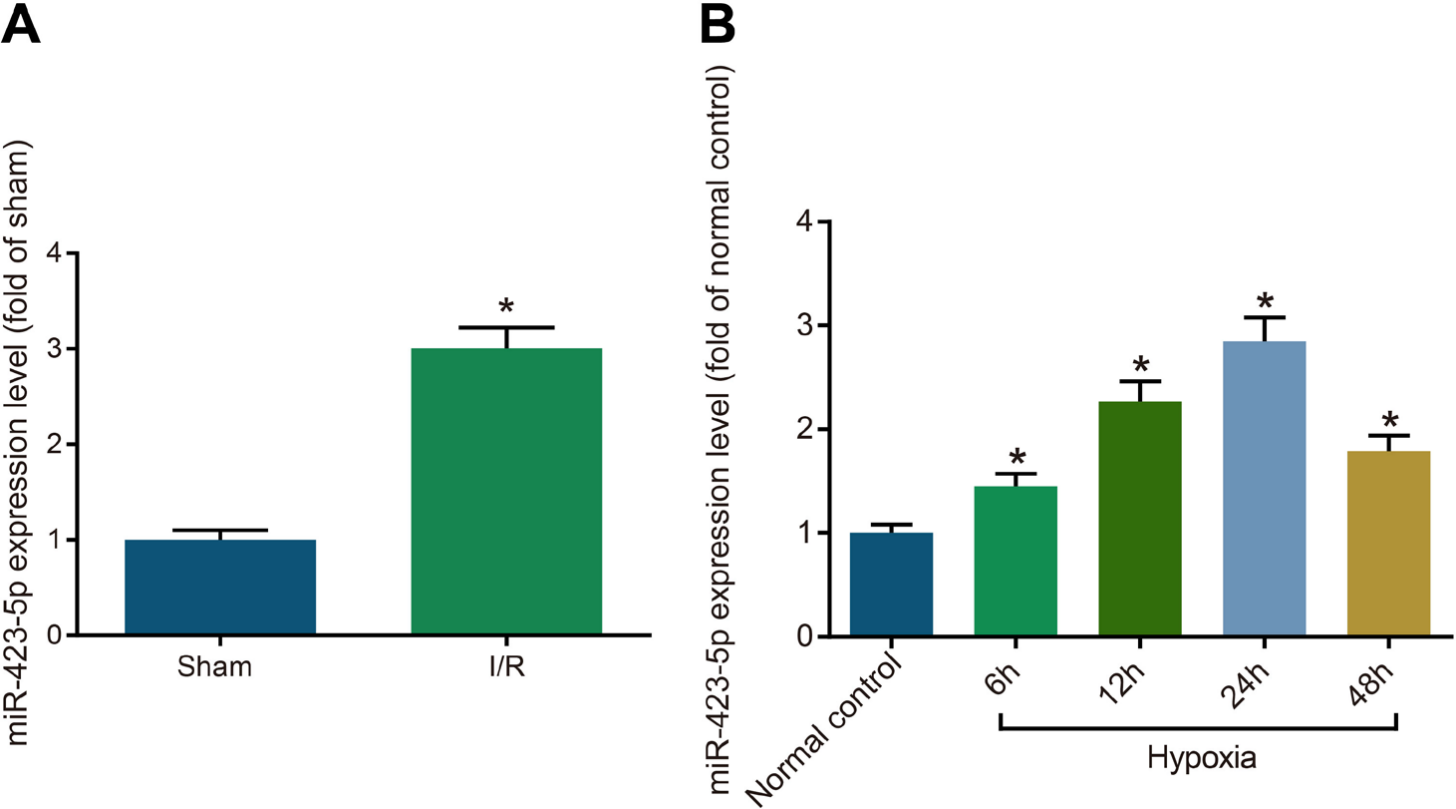
Increased miRNA-423-5p expression in kidney tissues after acute I/R and hypoxia/reoxygenation induced HK-2 cells (**A**) Estimation of miRNA-423-5p levels in kidney tissues of sham and I/R rats by qRT-PCR. (**B**) Analysis of miRNA-423-5p levels in normoxia and hypoxia HK-2 cells by qRT-PCR. Note: *denotes *P* < 0.05 in comparison with sham or normal control group; I/R, ischemia/reperfusion; qRT-PCR, quantitative real-time polymerase chain reaction; miR-423-5p, microRNA-423-5p.

Renal tissues from the I/R group rats demonstrated higher miRNA-423-5p levels compared to sham rats (*P* < 0.05; Figure 1A). In regard to HK-2 cells, the H/R group showed higher miRNA-423-5p expression compared to the normal control group (*P* < 0.05) with the highest miRNA-423-5p expression at 24 h (Figure 1B). This suggested that miRNA-423-5p played a role in renal injury. We selected the 24 h hypoxia group for further functional experiments.

### Dual luciferase reporter analysis of miR-423-5p binding to GSTM1 3′UTR

**Figure 2:**
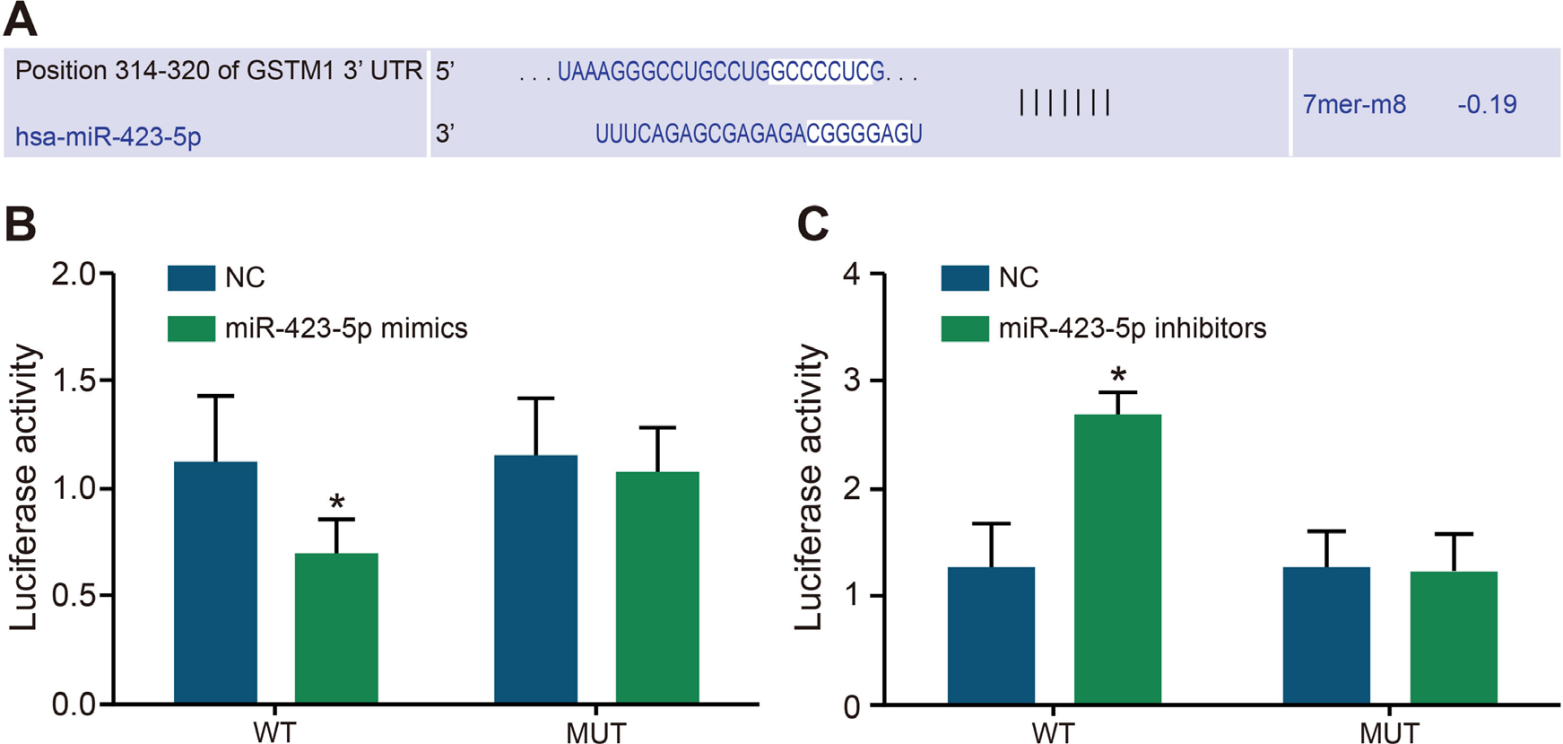
Dual luciferase assay analysis of miR-423-5p binding to GSTM1 3′UTR (**A**) Predicting miR-423-5p binding sites in GSTM1 sequence by miRBase. (**B**) Relative luciferase activity in HK-2 cells after co-transfection with miR-423-5p mimics and pGL3-GSTM1-WT or pGL3- GSTM1-MUT. (**C**) Relative luciferase activity in HK-2 cells after co-transfection with miR-423-5p inhibitors and pGL3-GSTM1-WT or pGL3- GSTM1-MUT. Note: *denotes *P* < 0.05 in comparison with the NC group; GSTM1, Glutathione-S-transferase (GST) M1; miR-423-5p, microRNA-423-5p; HK-2 cells, human renal tubular epithelial cells; NC, negative control.

The online bioinformatics software (miRBase Prediction Algorithm) was used to predict target genes which may be regulated by miR-423-5p. The result showed that the specific binding site of miR-423-5p was presented in the sequence of GSTM1 3′UTR (Figure 2A). Therefore, as described in the methods, we constructed the GSTM1 luciferase reporter vector to analyze the specific binding between miR-423-5p and GSTM1 3 ‘UTR. The dual luciferase reporter assay showed that relative luciferase activity decreased in H/R induced HK-2 cells after co-transfection of pGL3-GSTM1-WT and miR-423-5p mimics (*P* < 0.05; Figure 2B). However, relative luciferase activity increased in H/R induced HK-2 cells co-transfected with pGL3-GSTM1-WT and miR-423-5p inhibitors (Figure 2C). On the other hand, H/R induced HK-2 cells co-transfected with pGL3-GSTM1-MUT vector and miR-423-5p mimics or miR-423-5p inhibitors demonstrated no change (Figure 2B, 2C). These results confirmed that miR-423-5p specifically bound to GSTM1-3′UTR.

### Inhibition of GSTM1 expression by miR-423-5p in H/R induced HK-2 cells

**Figure 3:**
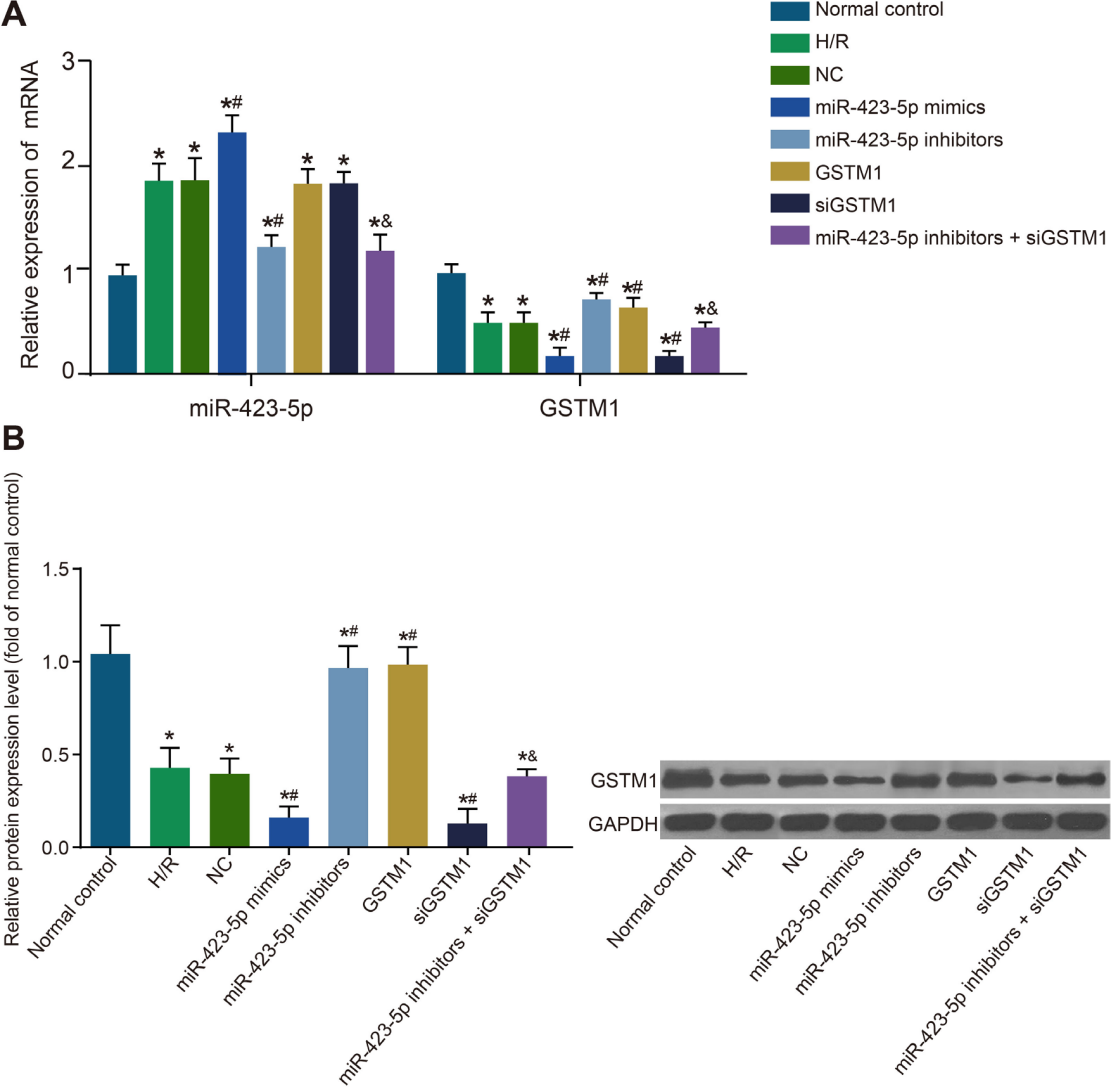
Analysis of miR-423-5p and GSTM1 expression in hypoxia/reoxygenation induced HK-2 cells (**A**) Levels of miR-423-5p and GSTM1 in (1) normal control, (2) H/R , (3) negative control, (4) miR-423-5p mimics, (5) miR-423-5p inhibitor, (6) GSTM1, (7) siGSTM1 and (8) miR-423-5p inhibitor plus siGSTM1 groups of HK-2 cells by qRT-PCR. (**B**) GSTM1 protein expression in HK-2 cells of the above 8 groups by western blotting. Note: *denotes *P* < 0.05 compared with the normal control group; ^#^denotes *P* < 0.05compared with the HR group; & denotes *P* < 0.05, compared with the miR-423-5p inhibitors group; miR-423-5p, microRNA-423-5p; GSTM1, Glutathione-S-transferase (GST) M1; HK-2 cells, human renal tubular epithelial cells; NC, negative control; H/R, hypoxia/reoxygenation.

The H/R induced HK-2 cells showed high miR-423-5p and lower GSTM1 mRNA and protein levels compared to the control group (both *P* < 0.05; Figure 3A–3B). However, expressions of miR-423-5p and GSTM1 in the H/R and NC groups were similar (both *P* > 0.05). The miR-423-5p expression in the miR-423-5p mimics group was upregulated compared to the H/R and NC groups whereas expression of GSTM1 mRNA and protein was down-regulated (all *P* < 0.05; Figure 3A–3B). In the miR-423-5p inhibitors group, miR-423-5p was downregulated and both GSTM1 mRNA and protein was up-regulated (all *P* < 0.05). In the GSTM1 group, the miR-423-5p expression was comparable to the NC group (*P* > 0.05), whereas GSTM1 mRNA and protein was up-regulated (all *P* < 0.05). In the siGSTM1 group, the miR-423-5p expression was similar to the NC group (*P* > 0.05), whereas GSTM1 mRNA and protein was down-regulated (all *P* < 0.05; Figure 3A–3B). In the miR-423-5p inhibitors + siGSTM1 group, GSTM1 mRNA and protein levels were down-regulated compared to the miR-423-5p inhibitors group (Figure 3A–3B). These results demonstrated that miR-423-5p negatively regulated GSTM1 expression.

### Inhibition of GSTM1 by miR-423-5p enhances ER stress in H/R induced HK-2 cells

**Figure 4:**
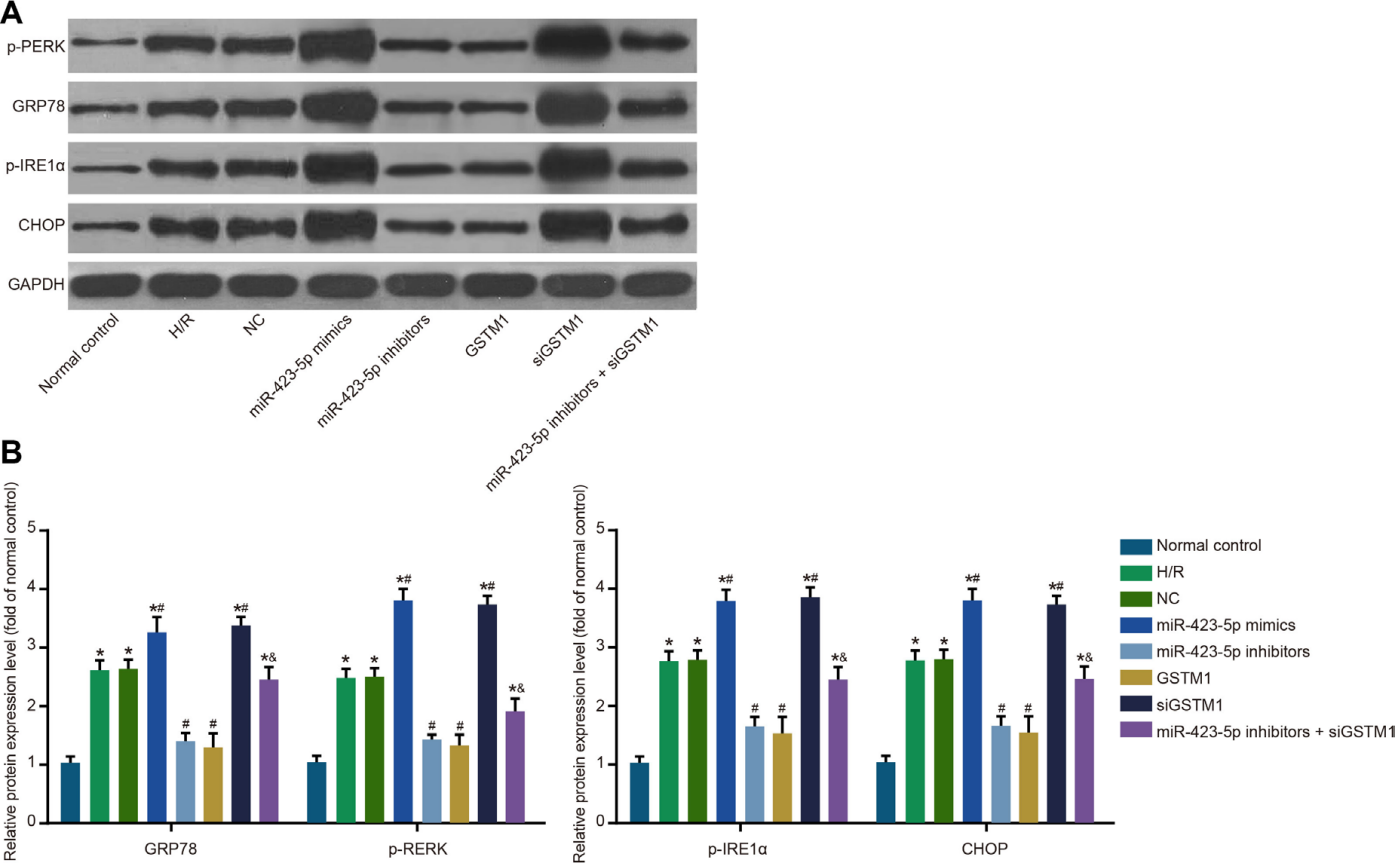
Analysis of ER stress in hypoxia/reoxygenation induced HK-2 cells (**A**) Analysis of ER-stress related proteins, GRP78, p-PERK, p-IRE1α and CHOP in (1) normal control, (2) H/R , (3) negative control, (4) miR-423-5p mimics, (5) miR-423-5p inhibitor, (6) GSTM1, (7) siGSTM1 and (8) miR-423-5p inhibitor plus siGSTM1 groups of H/R induced HK-2 cells by western blotting; (**B**) Relative levels of GRP78, p-PERK, p-IRE1α and CHOP proteins in the 8 experimental groups. Note: *denotes *P* < 0.05 compared with the normal control group; # denotes *P* < 0.05 compared with H/R group; & denotes *P* < 0.05 compared with miR-423-5p inhibitors group; miR-423-5p, microRNA-423-5p; GSTM1, Glutathione-S-transferase (GST) M1; ER, endoplasmic reticulum; GRP78, 78 kDa glucose-regulated protein; CHOP, C/EBP homology protein; NC, negative control; H/R, hypoxia/reoxygenation.

We observed increased ER stress-related proteins, namely GRP78, p-PERK, p-IRE1α and CHOP in the H/R group compared to control group (both *P* < 0.05; Figure 4B). The expression of ER stress-related proteins was similar in the H/R and NC groups (all *P* > 0.05). In the miR-423-5p mimics + siGSTM1 group, GRP78, p-PERK, p-IRE1α and CHOP expression was up-regulated, whereas in the miR-423-5p inhibitors + GSTM1 group, their expression was down-regulated (all *P* < 0.05). The levels of GRP78, p-PERK, p-IRE1α and CHOP were higher in the miR-423-5p inhibitors + siGSTM1 group compared to the miR-423-5p inhibitors group (all *P* < 0.05; Figure 4B). These results showed that inhibition of GSTM1 expression by miR-423-5p enhanced ER stress under H/R conditions.

### Inhibition of GSTM1 by miR-423-5p enhances oxidative stress in H/R induced HK-2 cells

**Figure 5:**
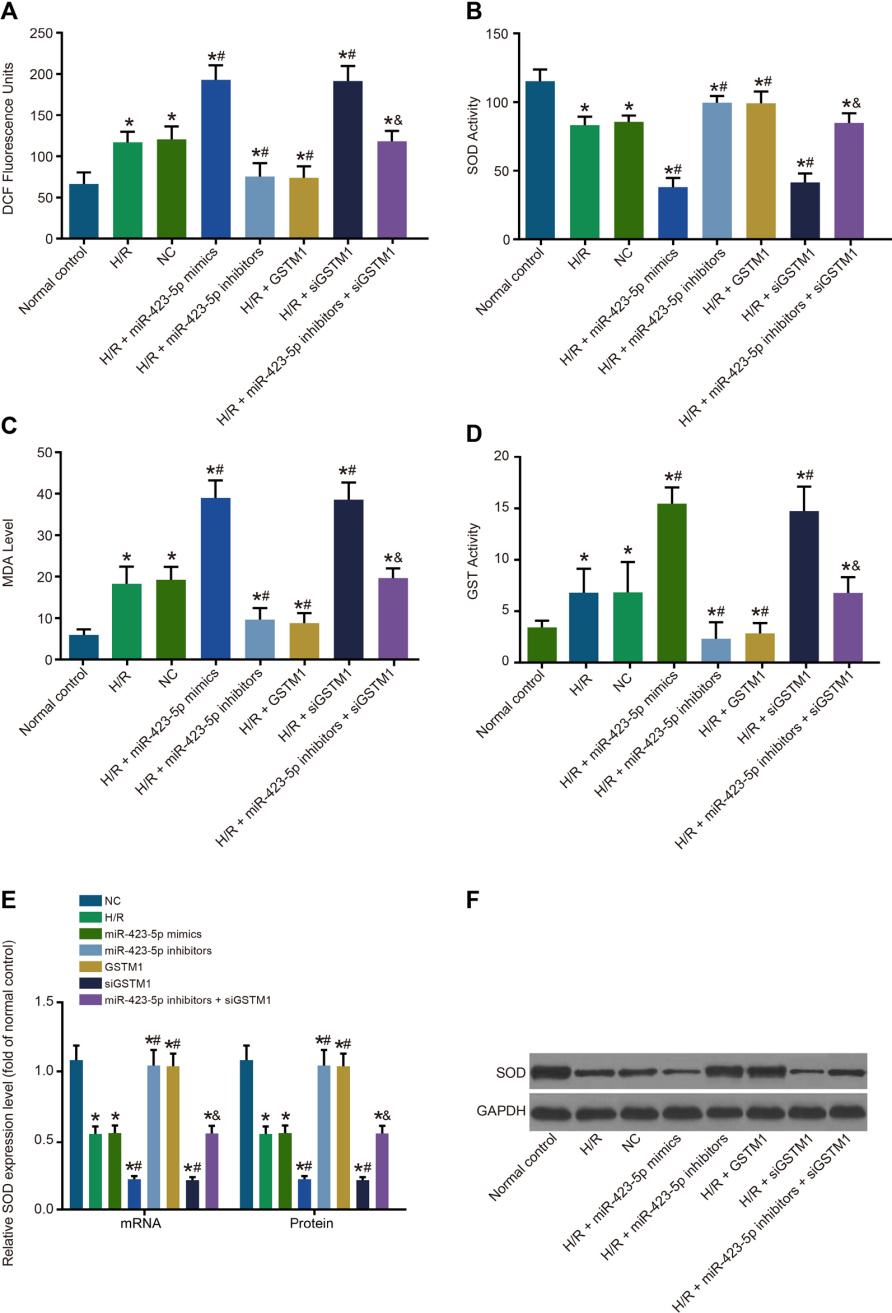
Analysis of oxidative stress in hypoxia/reoxygenation induced HK-2 cells (**A**) FACS analysis of cellular ROS in (1) normal control, (2) H/R, (3) negative control, (4) miR-423-5p mimics, (5) miR-423-5p inhibitor, (6) GSTM1, (7) siGSTM1 and (8) miR-423-5p inhibitor plus siGSTM1 groups of H/R induced HK-2 cells by intracellular DCHF-DA staining. (**B**) Analysis of cellular SOD activity in the 8 experimental HK-2 cell groups. (**C**) Analysis of cellular MDA activity in the 8 experimental HK-2 cell groups. (**D**) Analysis of cellular GST activity in the 8 experimental H/R induced HK-2 cell groups. (**E**) Expressions of SOD mRNA and protein in each group. (**F**) Protein band of SOD in each group by western blotting. Note: *denotes *P* < 0.05 compared with the normal control group; ^#^denotes *P* < 0.05 compared with the H/R group; & denotes *P* < 0.05 compared with the miR-423-5p inhibitors group; miR-423-5p, microRNA-423-5p; GSTM1, Glutathione-S-transferase (GST) M1; RPTEC, renal proximal tubular epithelial cell; NC, negative control; H/R, hypoxia/reoxygenation; ROS, reactive oxygen species; SOD, superoxide dismutase; MDA, malondialdehyde; GST, glutathione S-transferase.

We observed increased ROS levels and MDA and GST activities and decreased SOD activity in the H/R group compared to the control group (*P* < 0.05; Figure 5A–5D). There were no differences in the NC and H/R groups of HK-2 cells in regard to ROS levels and MDA, GST and SOD activities (all *P* > 0.05). In the miR-423-5p mimics and siGSTM1 groups, ROS levels and MDA and GST activities were up-regulated, whereas SOD activity was downregulated (all *P* < 0.05). Conversely, ROS levels and MDA and GST were down-regulated, whereas SOD activity was up-regulated in the miR-423-5p inhibitors and GSTM1 groups (all *P* < 0.05). The ROS levels and MDA and GST activities were upregulated, whereas SOD activity was downregulated in the miR-423-5p inhibitors + siGSTM1 group compared to miR-423-5p inhibitors group (all *P* < 0.05; Figure 5).

SOD mRNA and protein were decreased in the H/R group compared with the normal control group. However, SOD mRNA and protein levels were similar in the normal control and H/R groups (*P* > 0.05) (Figure 5E–5F). SOD mRNA and protein expressions were lower in the miR-423-5p mimics group compared to the miR-423-5p inhibitors group (*P* < 0.05). Also, SOD mRNA and protein expression were upregulated in the GSTM1 group, but downregulated in the siGSTM1 group (all *P* < 0.05). The SOD mRNA and protein levels were lower in the miR-423-5p inhibitors + siGSTM1 group compared to the miR-423-5p inhibitors group (*P* < 0.05; Figure 5E–5F). These results demonstrated that miR-423-5p inhibition of GSTM1 promote oxidative stress in H/R conditions.

### Inhibition of GSTM1 by miR-423-5p decreases cell proliferation in H/R induced HK-2 cells

**Figure 6:**
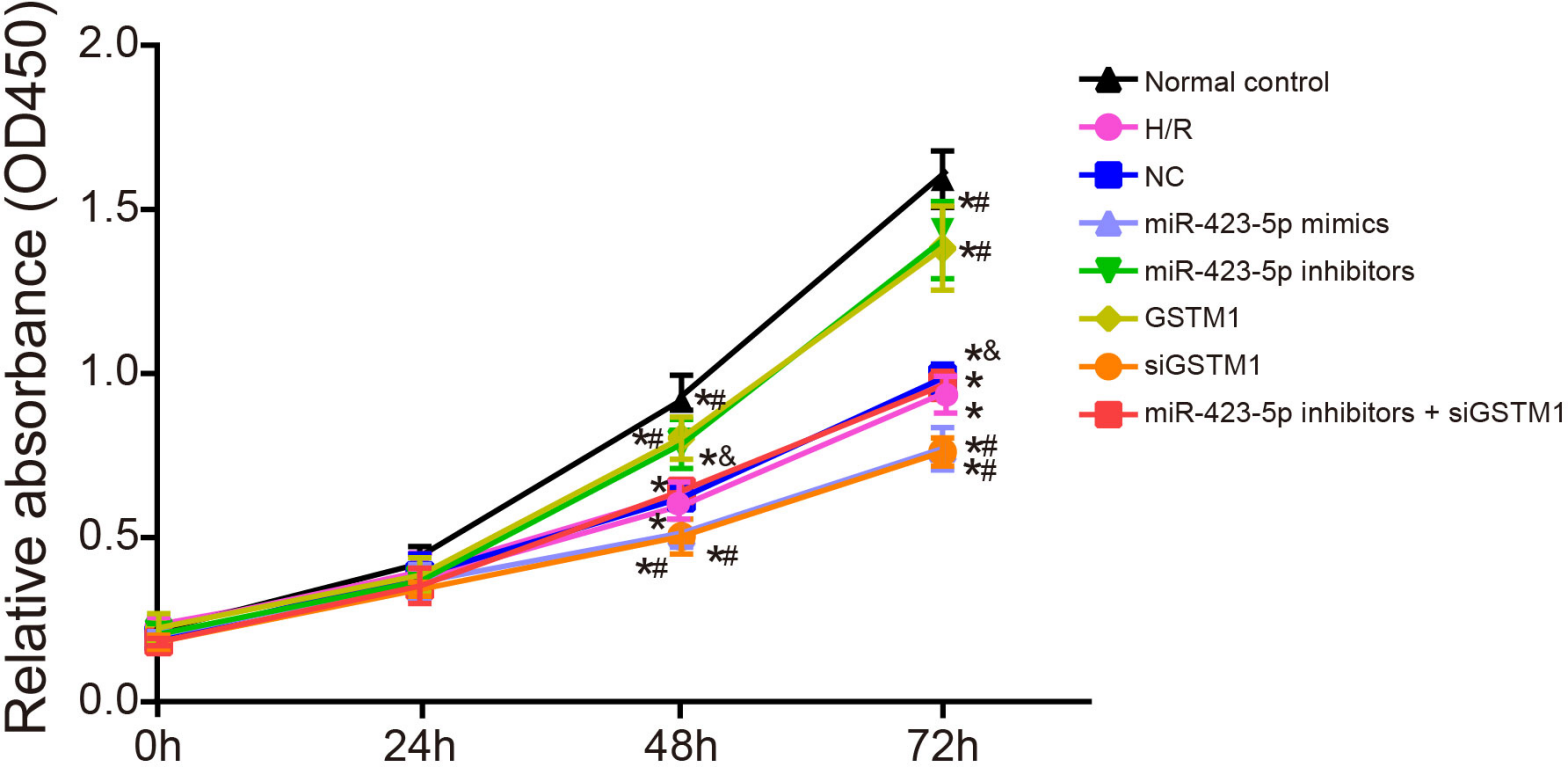
Analysis of proliferation in hypoxia/reoxygenation induced HK-2 cells by CCK-8 assay Estimation of proliferation in (1) normal control, (2) H/R, (3) negative control, (4) miR-423-5p mimics, (5) miR-423-5p inhibitor, (6) GSTM1, (7) siGSTM1 and (8) miR-423-5p inhibitor plus siGSTM1 groups of H/R induced HK-2 cells by CCK-8 assay. Note: *denotes *P* < 0.05 compared with the normal control; # denotes *P* < 0.05 compared with the H/R group; & denotes compared with the miR-423-5p inhibitors group; miR-423-5p, microRNA-423-5p; GSTM1, Glutathione-S-transferase (GST) M1; RPTEC, renal proximal tubular epithelial cell; NC, negative control; H/R, hypoxia/reoxygenation.

As shown in Figure 6, cell proliferation in all 8 groups was similar at the 24 h, but decreased in all groups in comparison to the normal control group at 48 and 72 h as determined by the CCK-8 assay. Cell proliferation was similar in H/R, NC and miR-423-5p inhibitors + siGSTM1 groups (*P* > 0.05). Cell proliferation was downregulated in the miR-423-5p mimics and siGSTM1 groups and upregulated in the miR-423-5p inhibitors and GSTM1 group compared to the H/R group (all *P* < 0.05). Also, cell proliferation in the miR-423-5p inhibitors + siGSTM1 group was lower than the miR-423-5p inhibitors group (Figure 6). These results showed that inhibition of GSTM1 by miR-423-5p decreased HK-2 cell proliferation in H/R conditions.

### Inhibition of GSTM1 by miR-423-5p increases cellular apoptosis in H/R induced HK-2 cells

**Figure 7:**
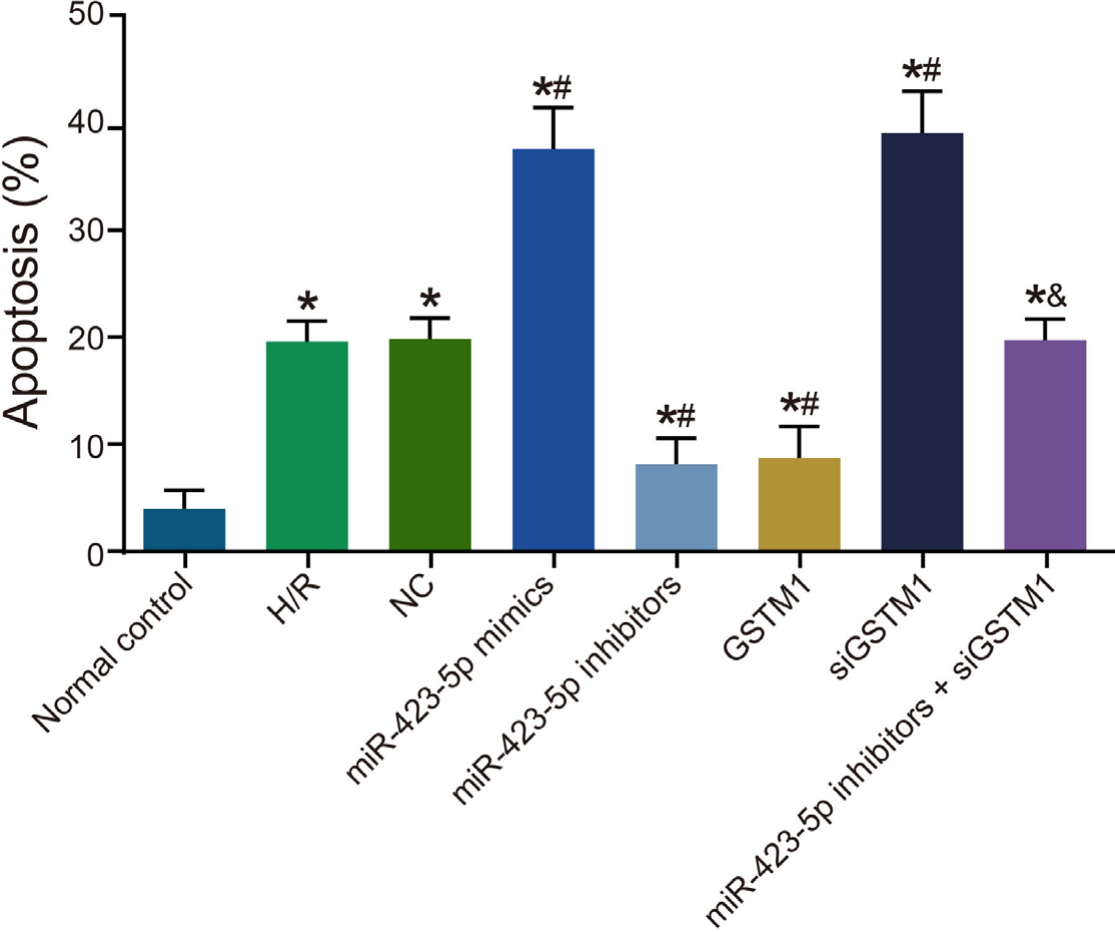
Analysis of cellular apoptosis in hypoxia/reoxygenation induced HK-2 cells FACS analysis of apoptosis in (1) normal control, (2) H/R, (3) negative control, (4) miR-423-5p mimics, (5) miR-423-5p inhibitor, (6) GSTM1, (7) siGSTM1 and (8) miR-423-5p inhibitor plus siGSTM1 groups of H/R induced HK-2 cells by AnnexinV/PI double staining. Note: * denotes *P* < 0.05 compared with the normal control group; # denotes *P* < 0.05 compared with the H/R group; & denotes compared with the miR-423-5p inhibitors group; ; miR-423-5p, microRNA-423-5p; GSTM1, Glutathione-S-transferase (GST) M1; RPTEC, renal proximal tubular epithelial cell; NC, negative control; H/R, hypoxia/reoxygenation.

Next, we analyzed the status of cellular apoptosis. We observed increased apoptosis in the H/R group compared to the normal control group at 24 h (Figure 7). Apoptotic rate was similar in the H/R, NC and miR-423-5p inhibitors + siGSTM1 groups (*P* > 0.05). In comparison to the H/R group, we observed increased apoptosis in the miR-423-5p mimics and siGSTM1 groups and decreased in the miR-423-5p inhibitors and GSTM1 groups (all *P* < 0.05; Figure 7). Also, higher rate of apoptosis was observed in the miR-423-5p inhibitors + siGSTM1 group than the miR-423-5p inhibitors group (*P* < 0.05). These results suggested that miR-423-5p inhibition of GSTM1 enhanced apoptosis in HK-2 cells apoptosis in H/R conditions.

### Renal function is reduced n I/R group rats

Rats subjected to I/R showed increased serum creatinine and urea nitrogen levels at day 1 that further increased at day 3 (both *P* < 0.05; Table 1). Although we observed that day 7 serum creatinine and urea nitrogen levels were lower than days 1 and 3 in the I/R treatment group, they were still higher than the sham group (both *P* < 0.05). The levels normalized earliest at day 14 or after day 21 (both *P* > 0.05; Table 1).

**Table 1:** Comparisons of serum creatinine and urea nitrogen levels in rats between the sham group and the I/R group (*n* = 7)

Group	Serum creatinine (μmol/L)	*P*	Urea nitrogen (mmol/L)	*P*
Sham group	31.13 ± 2.85		5.76 ± 0.71	
I/R group				
1 d	318.32 ± 14.67	*P* < 0.001	33.64 ± 8.16	*P* < 0.001
3 d	337.41 ± 12.63	*P* < 0.001	78.07 ± 7.19	*P* < 0.001
7 d	214.05 ± 17.43	*P* < 0.001	43.51 ± 3.24	*P* < 0.001
14 d	33.27 ± 6.28	0.24	6.15 ± 1.38	0.34
21 d	32.01 ± 8.69	0.71	5.89 ± 2.13	0.82

Note: I/R, ischemia/reperfusion.

### Expression of miR-423-5p and GSTM1 in I/R rat kidney

**Figure 8:**
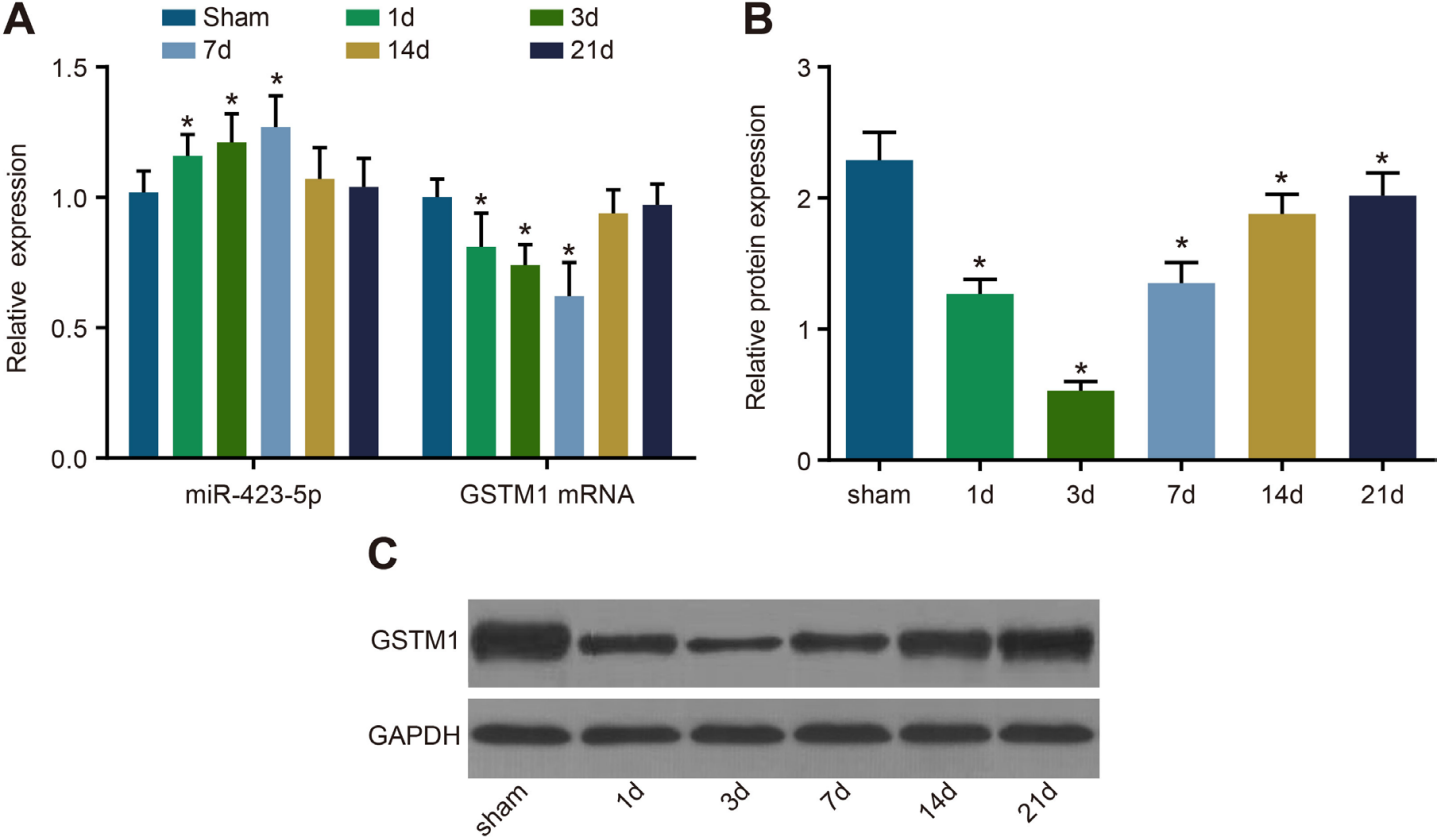
Analysis of miR-423-5p and GSTM1 levels in sham and I/R rat kidneys (**A**) Estimation of miR-423-5p and GSTM1 mRNA levels in the kidneys of sham and I/R group rats on days 1, 3, 7, 14 and 21 by qRT-PCR. (**B**) Quantification of GSTM1 protein levels in the renal tissues of sham and I/R group rats on days 1, 3, 7, 14 and 21 by analyzing gray values of western blots. (**C**) Western blotting analysis of GSTM1 in the renal tissues of sham and I/R group rats on days 1, 3, 7, 14 and 21 by western blotting. Note: *denotes *P* < 0.05 compared with the sham group; qRT-PCR, quantitative real-time polymerase chain reaction; miR-423-5p, microRNA-423-5p; GSTM1, Glutathione-S-transferase (GST) M1; RPTEC, renal proximal tubular epithelial cell; NC, negative control; I/R, ischemia/reperfusion.

We observed that miR-423-5p levels were up-regulated and GSTM1 mRNA and protein levels were down-regulated in I/R rats on days 1, 2 and 7 compared to the sham control group (all *P* < 0.05; Figure 8). Further, the levels of miR-423-5p and GSTM1 mRNA and protein normalized on days 14 and 21 (all *P* > 0.05; Figure 8).

### Effect of miR-423-5p mimics and inhibitors on renal function of I/R rats

Next, we analyzed the effect of miR-423-5p mimics and inhibitors on the renal function of I/R rats. We observed that on day 3, high serum creatinine and urea nitrogen levels in the I/R and I/R + miR-423-5p mimics group and lower serum creatinine and urea nitrogen levels in the I/R + miR-423-5p inhibitors group compared to the sham group (all *P* < 0.05; Table 2). This demonstrated that renal function improved upon miR-423-5p inhibition in I/R rats.

**Table 2:** Comparisons of serum creatinine and urea nitrogen levels in rats of each group (*n* = 15)

Group	serum creatinine (μmol /L)	*P*	Urea nitrogen (mmol /L)	*P*
Sham group	31.13 ± 2.85		5.76 ± 0.71	
I/R group	338.2 ± 8.11	*P* < 0.001	78.42 ± 7.59	*P* < 0.001
I/R + miR-423-5p mimic group	351.53 ± 6.23	0.040	92.04 ± 3.61	*P* < 0.001
I/R + miR-423-5p inhibitor group	312.2 ± 9.27	0.005	65.75 ± 5.64	*P* < 0.001

Note: I/R, ischemia/reperfusion; miR-423-5p, microRNA-423-5p.

### Effect of miR-423-5p on GSTM1 expression in I/R rat kidneys

**Figure 9:**
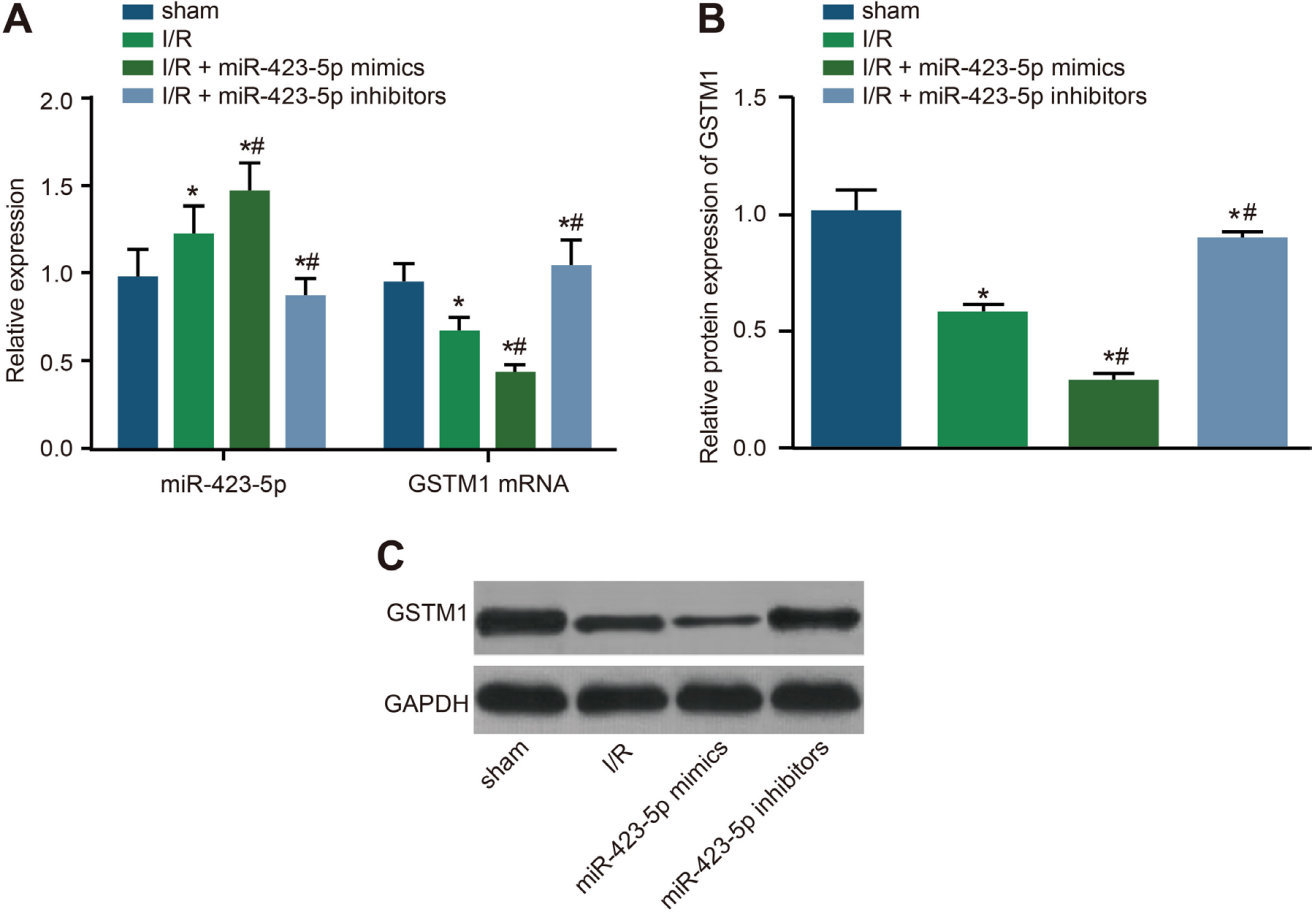
Effects of miR-423-5p mimics and inhibitors on GSTM1 expression in I/R treated rat kidneys (**A**) Expression miR-423-5p and GSTM1 mRNA in the renal tissues of I/R, I/R plus miR-423-5p mimics and I/R plus miR-423-5p inhibitors on day 3 by qRT-PCR. (**B**) Quantification of GSTM1 protein levels in the renal tissues of I/R, I/R plus miR-423-5p mimics and I/R plus miR-423-5p inhibitor groups by analysis of gray values in the western blotting. (**C**) Western blot analysis of GSTM1 protein expression in the renal tissues in I/R, I/R plus miR-423-5p mimics and I/R plus miR-423-5p inhibitors on day 3 after I/R. Note: *denotes *P* < 0.05 compared with the sham group; ^#^denotes *P* < 0.05 compared with the I/R group, *P* < 0.05; qRT-PCR, quantitative real-time polymerase chain reaction; miR-423-5p, microRNA-423-5p; GSTM1, Glutathione-S-transferase (GST) M1; I/R, ischemia/reperfusion.

Next, we compared the status of GSTM1 mRNA and protein in relation to miR-423-5p levels in the rat kidneys. On day 3, we observed higher miR-423-5p levels in the rat kidneys of I/R and I/R + miR-423-5p mimics group and lower miR-423-5p levels in the I/R + miR-423-5p inhibitors group compared to the sham group (all *P* < 0.05; Figure 9). Further, on day 3, GSTM1 mRNA and protein levels were lower in the I/R and I/R + miR-423-5p mimics groups and higher in the I/R + miR-423-5p inhibitors group compared to the sham group (all *P* < 0.05; Figure 9). This suggested that renal I/R induced miR-423-5p, which subsequently down-regulated GSTM1 expression.

## DISCUSSION

With a rat model of H/R and a rat model of I/R injury, the study confirmed the functions of miR-423-5p in the processes of RPETC injury by comparing the expressions and the changes of oxidative stress and ER stress after miR-423-5p treatment. Also, RPTECs are a vital factor in kidney function and miR-423-5p can regulate renal response to acute injury essential in maintenance of renal function and development of renal injury [2, 12]. Therefore, our study aims to explore the miR-423-5p effect on RPTECs.

The dual-luciferase reporter assay showed that miR-423-5p bound to the 3′UTR of *GSTM1* and suppressed its expression. GSTM1 belongs to one of the 5 Mu (μ) classes of GSTs in humans and is the most abundant among all GSTs in the kidneys of mice. However, approximately 30% to 50% of humans exhibit deficiency in the GSTM1 enzyme [18]. GSTM1 and glutathione-S transferase θ1 (GSTT1) are two predominant enzymes that detoxify and regulate oxidative stress [19]. GSTs detoxify the genetoxic metabolites into more water-soluble and readily execrable forms and thereby prevent ROS-induced membrane lipid peroxidation [20]. Our study confirmed that H/R injury of RPETCs induced miR-423-5p, which down-regulated expression of its target gene, GSTM1.

To further explore the consequences of and the negative regulation of GSTM1 by miR-423-5p in RPETC injury, we analyzed of the status of ER stress and oxidative stress. In the H/R injury model of RPETCs, miR-423-5p mimics and GSTM1 siRNA treatments increased ER stress and oxidative stress, whereas miR-423-5p inhibitors suppressed them. Also, when miR-423-5p was inhibited, cell proliferation increased and cellular apoptosis diminished. Since unresolved ER stress can lead to cell death, UPR stress sensors are a vital in determining cell fate (survival or apoptosis) [21]. The GRP78 and CHOP proteins are closely related to ER stress. GRP78 is constitutively expressed in the distal tubules of kidneys and binds to UPR sensors to repress the activities of CHOP and caspase-12 [22, 23]. Our study showed that the miR-423-5p inhibitors reduced GRP78 and CHOP expression and increased GSTM1 expression. This suggested that miR-423-5p increased ER stress in RPETCs by negatively regulating GSTM1.

SOD and MDA are critical markers of oxidative stress, whereas ROS influence cellular damage and renal dysfunction by inducing oxidative stress [24]. In a recent study, GSTM1 was associated with MDA and ROS, and loss of GSTM1 enhanced oxidative stress [16]. In this study, cells treated with miR-423-5p mimics demonstrated enhanced ROS and MDA and GST activities, whereas SOD activity was reduced. Since SOD is an antioxidant enzyme that acts against superoxide, decreased SOD activity suggested increased oxidative stress and decreased antioxidant capacity [25]. This trend was reversed by treating with miR-423-5p inhibitors that increased GSTM1. These results further showed that negative regulation of GSTM1 by miR-423-5p promoted oxidative stress during renal cell injury. Consistent with our data, another study showed that miR-423-5p inhibitors in gastric cancer cells reduced cell proliferation and increased tumor cell invasion [26]. In addition, miR-423-5p negatively regulated autophagy and cell cycle in hepatocellular carcinoma cells [27]. In this study, miR-423-5p increased RPETC apoptosis and decreased cell proliferation. This suggested that negative regulation of GSTM1 by miR-423-5p influences cell proliferation and apoptosis during renal injury.

In conclusion, our study demonstrates that miR-423-5p negatively regulates GSTM1 during renal cell injury, thereby affecting the repair process by enhancing oxidative stress and ER stress. Therefore, miR-423-5p inhibitors can be potentially therapeutic for RPTEC injury and need to be investigated.

## MATERIALS AND METHODS

### Ethical statement

All procedures were conducted strictly in conformity with the international guidelines and principles for Laboratory Animal Care.

### Cell culture

Human RPTECs (HK-2 cells) were obtained from American Type Culture Collection (ATCC) and cultured in RPMI1640 medium (Corning Glass Works, Corning, N.Y., USA) containing 10% fetal calf serum (Gibco, New York, NY, USA) at 37°C and 5% CO_2_ (LabServ CO-150, Thermo Fisher Scientific Inc., Waltham, MA, USA). When they reached confluence in 3–4 days, the cells were trypsinized (0.25% trypsin-EDTA; Sigma-Aldrich, Co. LLC, USA) and further sub-cultured or used for experiments [28].

### Dual luciferase reporter assay

The miRBase software was used to predict the binding sites of miR-423-5p in the 3′ UTR with the binding site of miR-423-5p was cloned into pGL3 vector (Promega Corp., Madison, Wisconsin, USA) and referred as pGL3-GSTM1-wild-type (WT). The miR-423-5p-binding site in the pGL3-GSTM1-WT plasmid was mutated to generate the pGL3-GSTM1-mutant-type (MUT) plasmid. Then, 5 × 10^4^/well HK-2 cells were grown in 24-well plates for 24 h at 37°C with 5% CO_2_ until they were 50 to 60% confluent. Then, we incubated the pGL3 firefly luciferase and the pRL renilla luciferase (Sigma Chemical Co, St Louis, USA) plasmids with the Lipofectamine 2000 transfection reagent for 15 min followed by transfecting them into the HK2 cells in the 24 well plates according to manufacturer’s instructions (Invitrogen Inc., Carlsbad, CA, USA). The medium was changed after 6 h, and the cells were cultured for an additional 48 h. The firefly luciferase and renilla luciferase activities in each transfection group were measured at 450 nm wavelength using a microplate reader system (GloMax, Promega, Madison, WI, USA) and the relative fluorescence calculated.

### HK-2 cell transfections and experimental grouping

Transfections were performed in HK-2 cells after hypoxia/reoxygenation (H/R) injury. The following were the 8 groups: (1) the normal control group without H/R treatment; (2) the H/R group (cells with H/R injury); (3) the negative control (NC) group (cells with H/R injury and transfected with nonsense sequence); (4) the miR-423-5p mimics group (cells with H/R injury and transfected with miR-423-5p mimics); (5) the miR-423-5p inhibitor group (with H/R injury and transfected with miR-423-5p inhibitors); (6) the GSTM1 group (cells with H/R injury and transfected with wild type GSTM1 gene); (7) the siGSTM1 group (cells with H/R injury and transfected with siRNA to GSTM1), and (8) the miR-423-5p inhibitor + siGSTM1 group (cells with H/R injury and transfected with miR-423-5p inhibitors and siRNA to GSTM1).

A nonsense sequence, miR-423-5p mimics and inhibitors, and siRNA to GSTM1 (siGSTM1) were purchased from GenePharma Co., Ltd (Shanghai, China). The miR-423-5p inhibitors are single-stranded RNA with chemical (2-methoxy) modification carried out after synthesis to ensure that specific binding with mature miR-423-5p would inhibit endogenous miR-423-5p expression. The pcDNA3.1-GSTM1 plasmid was purchased from Addgene (Cambridge, MA, USA). The transfection sequences are presented in Table 3. The transfected cells were plated at 50–60% confluence in 6-well plates at a density of 2 × 10^5^/well for 24 h at 37°C with 5% CO_2_. Lipofectamine™ 2000 transfection reagent (Thermo Fisher Scientific, Ltd., USA) was used to transfect either 50 nM nonsense sequence or 50 nM miR-423-5p mimics, 100 nM miR-423-5p inhibitors, 4 μg pcDNA3.1-GSTM1 plasmids. Lipofectamine™ RNAimax (Thermo Fisher Scientific, Ltd., USA) was used to transfect 50 nM siGSTM1. The medium was changed 6 h after transfection.

**Table 3:** Transfection sequences and qRT-PCR primers

Type	Sequence (5′–3′)
miR-423-5p (nonsense sequence)	CAGUACUUUUGUGUAGUA
miR-423-5p mimics	UGAGGGGCAGAGAGCGAGACUUU
miR-423-5p inhibitors	AAAGUCUCGCUCUCUGCCCCUCA
siGSTM1	CUCAAGCUAUGAGGAAAAGAAGU
miR-423-5p primers	F: 5′- ATGGTTCGTGGGTGAGGGGCAGAGAGCGAGAGCA
	GGGTCCGAGGTATTCG -3′
	R: 5′- GTGCAGGGTCCGAGGT -3′
GSTM1 primers	F: 5′- TTCCCAATCTGCCCTACTTG -3′
	R: 5′-TCTCCTCTTCTGTCTCCCCA -3′
U6 primers	F: 5′- CTCGCTTCGGCAGCACA -3′
	F: 5′- AACGCTTCACGAATTTGCGT -3′
SOD	F: 5′- AATGTGTCCATTGAAGATCGTGTGA -3′
	R: 5′-GCTTCCAGCATTTCCAGTCTTTGTA -3′
GAPDH	F: 5′- GACAACTTTGGCATCGTGGA -3′
	R: 5′-ATGCAGGGATGATGTTCTGG -3′

Note: qRT-PCR, quantitative real-time polymerase chain reaction; miR-423-5p, microRNA-423-5p; GSTM1, Glutathione-S-transferase (GST) M1; SOD, superoxide dismutase; GAPDH, glyceraldehyde phosphate dehydrogenase.

### Quantitative real-time polymerase chain reaction (qRT-PCR)

To detect miR-423-5p and GSTM1 mRNA levels by qRT-PCR, RNA was extracted from the 8 groups of experimental HK-2 cells or the rat kidneys according to the manufacturer’s instructions (Promega Corp., Madison, Wisconsin, USA). Equal amounts of RNA (1 μg) were reverse-transcribed to cDNA. Primer 5.0 software was used to design primers based on the gene sequences published in Genebank database. The primers are shown in Table 3.All primers were synthesized by Sangon Biotech Co., Ltd. (Shanghai, China). The real time PCR mix included 10 μL of 2 × SYBR Green mix (Roche, Switzerland) , 0.3 μL of 20 μM forward and 20 μM reverse primers each, 1 μL cDNA and 8.4 μL ddH_2_O. The real time PCR protocol was 95°C for 5 mins followed by 40 cycles of 95°C for 30 sec and 60°C for 1 min conducted in Applied Biosystems ViiA™ 7 Real-Time PCR instrument (Thermo Fisher Scientific, Ltd., USA). The relative expressions of miRNA-423-5p and GSTM1 mRNA were determined in relation to U6 and glyceraldehyde-3-phosphate dehydrogenase (GAPDH), respectively. For quantification, the relative expression levels of miRNA-423-p and GSTM1 mRNA in the normal control and sham groups were set to 1, respectively.

### Construction of the RPTEC injury model

To model RPTEC injury in cells, logarithmically growing HK-2 cells were washed twice with PBS and incubated in PBS in a 1% O_2_ + 94% N_2_ + 5% CO_2_ incubator (LabServ CO-150, Thermo Fisher Scientific Inc., Waltham, MA, USA). They were then incubated in hypoxic conditions (without O_2_) for 4 h at 37°C and then re-oxygenated. Then, PBS was replaced by normal medium and the cells were placed in a 5% CO_2_ + 95% air incubator for 12 h at 37°C.

### Western blotting

The concentration of the extracted protein was measured with the bicinchoninic acid (BCA) reagent kit (Beyotime Biotechnology Co., Shanghai, China). Equal amounts (30 μg) of total protein was mixed with SDS loading buffer and boiled for 10 min at 100°C and resolved on 10% SDS-PAGE for 30 min at 80V and then at 120V for 1h. Then, the proteins were wet transferred onto a PVDF membrane for 90 min at 100V. The membrane was blocked in 5% BSA (Beyotime Biotechnology Co., Shanghai, China) at room temperature for 1h, and then probed with primary antibodies against GSTM1 (ab113432, 1: 1000), 78 kDa glucose-regulated protein (GRP78) (ab173613, 1: 100), p-PERK (CST, #3179, 1: 1000), p-IRE1α (NB100-2323, 1: 1000) and C/EBP homology protein (CHOP) (ab10444, 1: 250), GAPDH (CST, #2118, 1: 1000) and superoxide dismutase (SOD) (ab13533, 1: 5000) overnight at 4°C. The membranes were then washed by TBST thrice for 5 mins and incubated with the corresponding secondary antibodies for 1h. The blots were then washed in TBST thrice for 5 min and developed with ECL chemiluminescence reagent (Nanjing Vazyme Biotech Co. Ltd, Nanjing, China) and imaged by the Bio-Rad Gel Dol EZ imager (GEL DOC EZ IMAGER, Bio-Rad, CA, USA). GAPDH was used as internal reference and the relative levels of different proteins were quantified.

### Analysis of oxidative stress

To measure reactive oxygen species (ROS) levels, cells in all HK-2 cell groups were trypsinized after 48 h. The cells were then incubated with DCFH-DA for 20 min at 37°C and after PBS washing and analyzed by flow cytometry (FACSCalibur, BD Biosciences, San Jose, CA, USA) with excitation at 488 nm and emission at 525 nm.

To determine superoxide dismutase (SOD) activity, cells were lysed with 100 μL of RIPA buffer on ice for 20 min and centrifuged for 30 min at 12,000 rpm. The supernatant was quantified by the BCA method. The SOD activity in all experimental groups was determined with the SOD activity assay kit (Shanghai Meilian Bioengineering Institute, Shanghai, China). The SOD activity was expressed as units/mg protein based on the protein concentration and dilution ratio of the samples.

To measure malondialdehyde (MDA) levels in cells, cell lysates were prepared as described for SOD activity. The samples were diluted to various concentrations (1, 2, 5, 10, 20, and 50 μM) with distilled water to prepare the standard curve. Then, 0.2 mL MDA detection fluid (Shanghai Meilian Bioengineering Institute, Shanghai, China) was added to eppendorf tubes with either 0.1-mL DDH_2_0 (as blank control) or 0.1-mL standard of different concentrations or 0.1mL cell lysates. The solutions were mixed and placed in a water bath for 15 min at room temperature and centrifuged at 1,000 rpm for 10 min. Then, 200 μL supernatant was added into a 224-well plate, and the absorbance determined at 532 nm (HBS-1096A, Nanjing Detie Experimental Equipment Company, Jiangsu, China). The molar concentrations of MDA in all samples were determined from the standard curve.

To measure glutathione S-transferase (GST) activity, cell lysates were prepared similar to SOD activity. The GST activity was determined using the GST assay kit (Shanghai Meilian Bioengineering Institute, Shanghai, China). Briefly, 190 μL reagent C was added to the 224-well plate followed by 25 μL reagent D and 10 μL reagent E. The plate was incubated for 3 mins at 25°C. Then, 25 μL reagent F (negative control liquid) or the sample to be tested (10 μg protein) was added to the corresponding wells. The absorbance at 340 nm was measured at 0min and 5 min. GST activity of sample = [(reading value of sample – reading value of background) × dilution ratio of sample × 0.25]/[0.025 × 5.03 × 0.6 × 5]/protein concentration of sample.

### Cell Counting Kit-8 (CCK-8) assay

Cell proliferation was determined by the CCK-8 assay kit (Nanjing Jiancheng biotech Co. Ltd., Jiangsu, China). Briefly, 10 μl CCK-8 reagent was added to transfected HK-2 cells at 0, 24, 48 and 72 h, respectively and incubated for 1–3h. The absorbance was detected at 450 nm. Each group was analyzed in triplicate. All experiments were performed in triplicates.

### Flow cytometry

To determine apoptosis by flow cytometry, transfected cells were trypsinized without EDTA after 48 h culturing and stained by AnnexinV-FITC and PI staining kit (Shanghai Qcbio Science & Technologies Co. Ltd., Shanghai, China). The stained cells were analyzed by flow cytometry (FACSCalibur, BD Biosciences, San Jose, CA, USA) and the percentage of AnnexinV^+^ PI^+^ cells in each group were determined. All experiments were performed in triplicate.

### Rat Model for renal injury

Ninety male SD rats (28 days old weighing 190–210 g) were obtained from Shanghai Experimental Animal Center (Shanghai, China). All animal feeding and operations were in accordance with experimental animal ethics and animal welfare. The rats were housed in the conservatory (20–28°C) with 12h/12h light/dark cycle with adequate drinking water and food. The animals were randomly divided into the sham (*n* = 15) and I/R groups (*n* = 75). The I/R group was further divided into five groups of 15 rats each representing days 1, 3, 7, 14, and 21 time points. The rats in the sham-control group were opened but did not receive any treatment after finding the kidney pedicles. For the I/R group, the kidney pedicles of rats were clamped for 60 min. Then, the arterial clamp was removed, and renal blood flow was allowed. After the operation, the rats were placed in the normal environment, and their vital signs observed.

### Evaluation of renal function

Serum samples were prepared from 2 ml blood obtained from the aorta abdominalis in rats by centrifuging at 3000 rpm for 5 min. Serum creatinine and urea nitrogen were determined from the serum of sham and the I/R group rats in the automatic biochemistry analyzer (Beckman Coulter, Inc., Brea, California, USA).

### Treatment of rats with miR-423-5p mimics or inhibitors

After I/R treatment of SD rats, miR-423-5p mimics or inhibitors were mixed in a 1: 2 ratio with Entranster™ (Engreen Biosystem Co. Ltd., Beijing, China) and intravenously injected through the rat tails. After 3 days, the relative index of renal function and miR-423-5p and GSTM1 expression were determined.

### Statistical analysis

All data were analyzed by SPSS 18.0 software (SPSS Inc., Chicago, IL, USA). The data were presented as the mean ± standard deviation (SD). The experimental groups were compared by *t* test and paired *t* test. Comparison among groups was analyzed by one-way analysis of variance (ANOVA), and statistical correlation was determined by the Pearson correlation test. The level of significance was set as *P* < 0.05.

## References

[1] Hou S, Zheng F, Li Y, Gao L, Zhang J (2014). The protective effect of glycyrrhizic acid on renal tubular epithelial cell injury induced by high glucose. Int J Mol Sci.

[2] Zhou TB, Qin YH, Lei FY, Huang WF, Drummen GP (2013). Prohibitin is associated with antioxidative protection in hypoxia/reoxygenation-induced renal tubular epithelial cell injury. Sci Rep.

[3] Hirose M, Yasui T, Okada A, Hamamoto S, Shimizu H, Itoh Y, Tozawa K, Kohri K (2010). Renal tubular epithelial cell injury and oxidative stress induce calcium oxalate crystal formation in mouse kidney. Int J Urol.

[4] Wu CT, Sheu ML, Tsai KS, Weng TI, Chiang CK, Liu SH (2010). The role of endoplasmic reticulum stress-related unfolded protein response in the radiocontrast medium-induced renal tubular cell injury. Toxicol Sci.

[5] Chandrika BB, Yang C, Ou Y, Feng X, Muhoza D, Holmes AF, Theus S, Deshmukh S, Haun RS, Kaushal GP (2015). Endoplasmic Reticulum Stress-Induced Autophagy Provides Cytoprotection from Chemical Hypoxia and Oxidant Injury and Ameliorates Renal Ischemia-Reperfusion Injury. PLoS One.

[6] Kawakami T, Inagi R, Takano H, Sato S, Ingelfinger JR, Fujita T, Nangaku M (2009). Endoplasmic reticulum stress induces autophagy in renal proximal tubular cells. Nephrol Dial Transplant.

[7] Baradaran A, Nasri H, Rafieian-Kopaei M (2015). Protection of renal tubular cells by antioxidants: current knowledge and new trends. Cell J.

[8] Anglicheau D, Muthukumar T, Suthanthiran M (2010). MicroRNAs: small RNAs with big effects. Transplantation.

[9] Hulsmans M, De Keyzer D, Holvoet P (2011). MicroRNAs regulating oxidative stress and inflammation in relation to obesity and atherosclerosis. FASEB J.

[10] Liu R, Zhang C, Hu Z, Li G, Wang C, Yang C, Huang D, Chen X, Zhang H, Zhuang R, Deng T, Liu H, Yin J (2011). A five-microRNA signature identified from genome-wide serum microRNA expression profiling serves as a fingerprint for gastric cancer diagnosis. Eur J Cancer.

[11] Tijsen AJ, Creemers EE, Moerland PD, de Windt LJ, van der Wal AC, Kok WE, Pinto YM (2010). MiR423-5p as a circulating biomarker for heart failure. Circ Res.

[12] Bruno N, ter Maaten JM, Ovchinnikova ES, Vegter EL, Valente MA, van der Meer P, de Boer RA, van der Harst P, Schmitter D, Metra M, O’Connor CM, Ponikowski P, Teerlink JR (2016). MicroRNAs relate to early worsening of renal function in patients with acute heart failure. Int J Cardiol.

[13] Shepperd JA, Lipkus IM, Sanderson SC, McBride CM, O’Neill SC, Docherty S (2013). Testing different communication formats on responses to imagined risk of having versus missing the GSTM1 gene. J Health Commun.

[14] Singh R, Manchanda PK, Kesarwani P, Srivastava A, Mittal RD (2009). Influence of genetic polymorphisms in GSTM1, GSTM3, GSTT1 and GSTP1 on allograft outcome in renal transplant recipients. Clin Transplant.

[15] Cheng HY, You HY, Zhou TB (2012). Relationship between GSTM1/GSTT1 null genotypes and renal cell carcinoma risk: a meta-analysis. Ren Fail.

[16] Chang J, Ma JZ, Zeng Q, Cechova S, Gantz A, Nievergelt C, O’Connor D, Lipkowitz M, Le TH (2013). Loss of GSTM1, a NRF2 target, is associated with accelerated progression of hypertensive kidney disease in the African American Study of Kidney Disease (AASK). Am J Physiol Renal Physiol.

[17] Mitchell AE, Morin D, Lakritz J, Jones AD (1997). Quantitative profiling of tissue- and gender-related expression of glutathione S-transferase isoenzymes in the mouse. Biochem J.

[18] Yang Y, Parsons KK, Chi L, Malakauskas SM, Le TH (2009). Glutathione S-transferase-micro1 regulates vascular smooth muscle cell proliferation, migration, and oxidative stress. Hypertension.

[19] Park EY, Hong YC, Lee KH, Im MW, Ha E, Kim YJ, Ha M (2008). Maternal exposure to environmental tobacco smoke, GSTM1/T1 polymorphisms and oxidative stress. Reprod Toxicol.

[20] Ito M, Muraki M, Takahashi Y, Imai M, Tsukui T, Yamakawa N, Nakagawa K, Ohgi S, Horikawa T, Iwasaki W, Iida A, Nishi Y, Yanase T (2008). Glutathione S-transferase theta 1 expressed in granulosa cells as a biomarker for oocyte quality in age-related infertility. Fertil Steril.

[21] Dufey E, Sepúlveda D, Rojas-Rivera D, Hetz C (2014). Cellular mechanisms of endoplasmic reticulum stress signaling in health and disease. 1. An overview. Am J Physiol Cell Physiol.

[22] Ohse T, Inagi R, Tanaka T, Ota T, Miyata T, Kojima I, Ingelfinger JR, Ogawa S, Fujita T, Nangaku M (2006). Albumin induces endoplasmic reticulum stress and apoptosis in renal proximal tubular cells. Kidney Int.

[23] Wang Y, Tian J, Qiao X, Su X, Mi Y, Zhang R, Li R (2015). Intermedin protects against renal ischemia-reperfusion injury by inhibiting endoplasmic reticulum stress. BMC Nephrol.

[24] Yu W, Sheng M, Xu R, Yu J, Cui K, Tong J, Shi L, Ren H, Du H (2013). Berberine protects human renal proximal tubular cells from hypoxia/reoxygenation injury via inhibiting endoplasmic reticulum and mitochondrial stress pathways. J Transl Med.

[25] Wu MM, Chiou HY, Wang TW, Hsueh YM, Wang IH, Chen CJ, Lee TC (2001). Association of blood arsenic levels with increased reactive oxidants and decreased antioxidant capacity in a human population of northeastern Taiwan. Environ Health Perspect.

[26] Liu J, Wang X, Yang X, Liu Y, Shi Y, Ren J, Guleng B (2014). miRNA423-5p regulates cell proliferation and invasion by targeting trefoil factor 1 in gastric cancer cells. Cancer Lett.

[27] Stiuso P, Potenza N, Lombardi A, Ferrandino I, Monaco A, Zappavigna S, Vanacore D, Mosca N, Castiello F, Porto S, Addeo R, Prete SD, De Vita F (2015). MicroRNA-423-5p Promotes Autophagy in Cancer Cells and Is Increased in Serum From Hepatocarcinoma Patients Treated With Sorafenib. Mol Ther Nucleic Acids.

[28] Zhao J, Wang L, Cao AL, Jiang MQ, Chen X, Wang Y, Wang YM, Wang H, Zhang XM, Peng W (2016). HuangQi Decoction Ameliorates Renal Fibrosis via TGF-β/Smad Signaling Pathway In Vivo and In Vitro. Cell Physiol Biochem.

